# Study Protocol of a Prospective Multicenter Study on Patient Participation for the Clinical Trial: Surgery as Needed Versus Surgery on Principle in Post-Neoadjuvant Complete Tumor Response of Esophageal Cancer (ESORES)

**DOI:** 10.3389/fonc.2021.789155

**Published:** 2022-01-18

**Authors:** Joachim Weis, Andrea Kiemen, Claudia Schmoor, Julian Hipp, Manuel Czornik, Matthias Reeh, Peter P. Grimminger, Christiane Bruns, Jens Hoeppner

**Affiliations:** ^1^ Endowed Professorship Self-Help Research, Comprehensive Cancer Center, Faculty of Medicine and Medical Center, University of Freiburg, Freiburg, Germany; ^2^ Clinical Trials Unit, Faculty of Medicine and Medical Center, University of Freiburg, Freiburg, Germany; ^3^ Department of General Surgery, Faculty of Medicine and Medical Center, University of Freiburg, Freiburg, Germany; ^4^ Department of General, Visceral and Thoracic Surgery, University Medical Center Hamburg-Eppendorf, Hamburg, Germany; ^5^ Department of General, Visceral and Transplantation Surgery, University Medical Center Mainz, Mainz, Germany; ^6^ Department of General, Visceral, Cancer and Transplantation Surgery, University Hospital of Cologne, Cologne, Germany; ^7^ Clinic for Surgery, University Medical Center Schleswig-Holstein, Lübeck, Germany

**Keywords:** patient participation, esophageal cancer (EC), patient-centered, study information, psycho-social needs, informed consent

## Abstract

Ideally, patient-centered trial information material encourages the discussion with the treating physician, and helps patients making trade-offs regarding treatment decisions In a situation of possible equivalent treatment options in terms of overall survival (OS), it can make it easier to weigh up advantages and disadvantages. Preferences for choice of treatment in esophageal cancer (EC) are complex, and no standardized assessment tools are available. We will explore patient’s factors for treatment choice and develop a comprehensive patient information leaflet for the inclusion into randomized controlled trials (RCT) on EC. We conduct a cross-sectional, observational study based on a mixed-methods design with patients suffering from non-metastatic EC with post-neoadjuvant complete response after neoadjuvant chemotherapy (nCT) or neoadjuvant chemoradiation (nCRT), to develop patient-centered trial information material. This pilot study is performed in a concept development phase and a subsequent pilot phase. We start with patient interviews (*n* = 10–15) in the concept development phase to evaluate patients’ needs, and develop a Preference and Decision Aid Questionnaire (PDAQ). We pre-test the PDAQ with another *n* = 10 patients with EC after nCT or nCRT, former patients from a self-help organization, and *n* = 10 medical experts for their comments on the questionnaire. In the pilot phase, a multicenter trial using the PDAQ and additional measures is carried out (*n* = 120). Based on evidence of a possible equivalence in terms of OS of the treatment options “surgery as needed” and “surgery on principle” in patients with post-neoadjuvant complete response of EC, this pilot study on patient participation is conducted to assess patient’s needs and preferences, and optimize patients’ inclusion in a planned RCT. The aim is to develop patient-centered trial information material for the RCT to increase patients’ consent and compliance with the randomized treatment. The trial is registered at the German Clinical Trials Register (DRKS00022050, October 15, 2020).

## Introduction

Patient-centered health care considers patients and professionals as partners and has its focus on the individual patient’s treatment preferences and needs. Thus, patients should be treated as partners with solidarity, empathy, and collaboration, but also with responsibilities (1). An essential point of a modern high-quality health care system is the treatment decision-making process, in which the patient is actively involved by getting relevant information in terms of treatment options. Treatment decision for patients with cancer is a complex task and requires a patient-oriented information process. In the process of providing information, it has to be considered that the patient is in a highly distressed situation in consequence of the diagnosis. Balancing of risks and benefits aimed to reach an understanding of the patient in a difficult situation of treatment options is an important challenge in the process of informed consent in clinical trials. This is regarded as essential also for the information process in randomized trials, and it is anticipated that it will improve the recruitment process and consent to randomization (2). Ideally, patient-centered trial information material encourages the discussion with the treating physician, and helps patients making trade-offs that reflect their own values and preferences (3). In consequence, patient-centered trial information material should include evidence-based information on disease and treatment options, postoperative mortality and morbidity, intermediate and long-term outcomes, side-effects, and burdens to daily life of respective treatment options (4). Educational material should be included, addressing risks and benefits of treatment options. The ethically optimal procedure is one that empowers patients to make preference-sensitive decisions consistent with their goals, values, and preferences (5). Even though shared decision making is not the envisaged process in randomized trials, value clarification is important. The decision to take part in a randomized clinical trial is driven by subjective and intuitive behavior [e.g., feeling of discomfort and vulnerability (2, 5, 6)] and has also psychological, social, and emotional factors (7). The tools of shared decision making processes are useful to gain a more comprehensive understanding of patients’ attitudes towards treatment and clinical trials in general, and to take this information into account in the study information material.

The standard of care for patients with non-metastatic esophageal cancer (EC) after neoadjuvant chemoradiotherapy (nCRT) or neoadjuvant chemotherapy (nCT) is principal surgery, 4–8 weeks after nCRT/nCT (8–10). Evaluations of health-related quality of life (HRQoL) for patients who had EC treated with nCRT showed detrimental effects in physical functioning, odynophagia, fatigue, weight loss, and global quality of life in those 4–6 weeks prior to surgery (11). Rapidly physical functioning, odynophagia, and sensory symptoms were restored to pretreatment levels respectively 4–10 weeks after nCRT (11). After surgery role and social function, fatigue, diarrhoea, appetite loss, nausea and vomiting became substantially worse compared to a reference population. Overall HRQL in long-term survivors after esophagectomy did not improve between 6 months and 3 years after surgery, and was worse than that in a comparable reference population (12).

Another as equivalent hypothesized treatment option following nCRT or nCT in terms of overall patient survival is close surveillance with surgery only as needed in persisting or recurring loco-regional tumor (13). A survival disadvantage of delayed surgery in case of local tumor relapse appears unlikely in a protocol of close surveillance of clinical complete response (cCR) (10, 14). Moreover, HRQoL can be restored to levels before treatment after 4–10 weeks after completion of nCRT (11). In a comparative analysis, 36 patients who underwent nCRT and surveillance were matched to 36 patients who underwent nCRT followed by direct surgery. Estimated median overall survival (OS) was equivalent in the surveillance group than in the standard surgery group (58 months, vs. 51 months, *p* = .28). All patients in the surveillance group with loco-regional recurrence in the absence of distant metastases underwent surgery as needed with excellent outcome (median OS 58 months). Moreover, distant metastasis rate was comparable in both groups (active surveillance: 31% vs. standard surgery: 28%) (10). Additionally, we conducted a systematic scoping review of all available studies on the comparison of “surgery as needed” versus “surgery on principle” (15). The results suggest that both post-neoadjuvant treatments are feasible to evaluate in a prospective and comparative clinical trial for complete clinical responders without compromising on OS. Thus, post-neoadjuvant identification of patients with pathological complete response (pCR) followed by closed-meshed surveillance and surgery as needed in case of local tumor recurrence might be a treatment alternative to surgery on principle for patients with post-neoadjuvant pCR. Before practice in routine clinical pathways this has to be evaluated by prospective randomized controlled trials (RCTs). Quality of life is expected to be clearly improved in this group of patients. Omission of esophagectomy reduces length of therapy, complication rate, and time of hospital stay resulting in a reduced treatment cost and faster return to socioeconomic productive work life of the patients.

Clinical response evaluation in the subsequent RCT comprises esophagogastroscopy to locate mucosal tumor, residual or scarred lesions, endoscopic deep biopsies of tumor area to obtain proof or exclusion of residual tumor, endoscopic ultrasound plus fine needle aspiration (FNA) of suspicious lymph nodes to proof or exclude of residual tumor, pathology workup of biopsies and FNA aspirates and F18-FDG-PET CT (whole body) for radiographic/metabolic targeting of loco-regional/distant disease. Clinical response evaluation is done 4-8 weeks after completion of neoadjuvant treatment. In case of clinical histology-proven positive tumor status and/or loco-regional metabolic positive lymph nodes without distant metastasis after clinical response evaluation (“non-CR”), treatment is surgery (Esophagectomy). Patients without histologic evidence of local residual disease, without loco-regional metabolic positive lymph nodes and without evidence for distant metastasis will be considered to be clinically complete responders (“clinical CR”) and will be to directly proceed with consecutive close-meshed surveillance visits with surgery only in the event of a local tumor recurrence. 

In the situation of possible equivalent treatment options in terms of OS, patient-centered trial information can make it easier to weigh up the advantages and disadvantages of the alternatives. This understanding is the prerequisite for an informed decision. Treatment options can be described by discrete attributes, and the value of the treatment options depends on the nature and level of these attributes. A prospective study showed that 5-year OS, long-term HRQoL, and the chance that esophagectomy is still necessary influenced patients’ preference for either active surveillance or planned surgery after nCRT for esophageal cancer (16). A study among patients who had undergone esophagectomy concluded that patients are willing to trade-off 16% of their 5-year survival chance to achieve an improvement in early outcomes (17). Using regression coefficients (β) as measures for the relative importance of attributes, a patient survey assessing preferences of patients towards surgery for preoperative esophagogastric cancer evaluated that patients preferred a better quality of life (QoL) (β = 1.19), higher cure rate (β = 0.82), and lower morbidity (β = 0.70) over treatment in a specialist hospital (β = 0.26) (4).

The factors influencing patients’ treatment preferences for choice of treatment in esophageal cancer are complex, and no standardized assessment tools are available.

### Aims and Purpose of the Study

The purpose of the study is to develop patient-centered trial information material to be used in the planned RCT designed to compare the treatment regimens “surgery as needed” and “surgery on principle” in patients with post-neoadjuvant complete response of EC with respect to OS. The aim of this study is to improve the recruitment of patients in the planned RCT and to improve their consent to randomization and their adherence to the randomized treatment. In a first step we will assess patients’ information needs and values in terms of the two treatment options of the envisaged RCT by qualitative interviews. Based on these results a Preference and Decision Aid Questionnaire (PDAQ) will be designed in the first part of the project (i.e., development phase). After pre-testing this questionnaire will be used in a survey in the second part of the project (i.e., pilot testing phase) for the evaluation of patients’ preferences and analysis of associations with fear of progression, depression, anxiety, health-related quality of life and disease related social support.

Further, the pilot phase will provide information on the likelihood of patients consenting to participation in the RCT, accepting randomization, and about their compliance with treatment allocation. Hereby we intend to improve the inclusion rate and to optimize the estimations on patient refusal rate, drop-out rate, and cross-over-rate of the envisaged RCT.

## Methods

In a cross-sectional, observational study we are assessing patients’ needs and preferences towards the treatment options of the planned RCT “Surgery as needed versus surgery on principle in patients with post-neoadjuvant complete tumor response of esophageal cancer (ESORES)” in two consecutive phases: (1) A development phase and (2) a pilot testing phase.

We start with detailed qualitative patient interviews (*n* = 10–15) in the development phase. Patients who had already undergone nCT or nCRT for EC and partially also surgery are asked for their needs, preferences, and attitudes towards choice of treatment. Particularly, patients are asked regarding their potential willingness to participate, to accept randomization, and to comply with the treatment to which they will be allocated.

Additionally, *n* = 10 medical experts in the field of EC treatment (i.e., 3–5 clinicians out of the field of EC treatment, 3–5 nurses, and psycho-oncologists) are asked regarding their experiences with patients in terms of patients’ attitudes towards treatment choices, preferred treatment option, and the reasons for it.

With respect to patient participation, *n* = 2 members of an adequate Peer-Support Organization (18) are asked to review the interview guidelines, the interview statements, and the provisional PDAQ, and to give comments on them.

**Figure 1:**
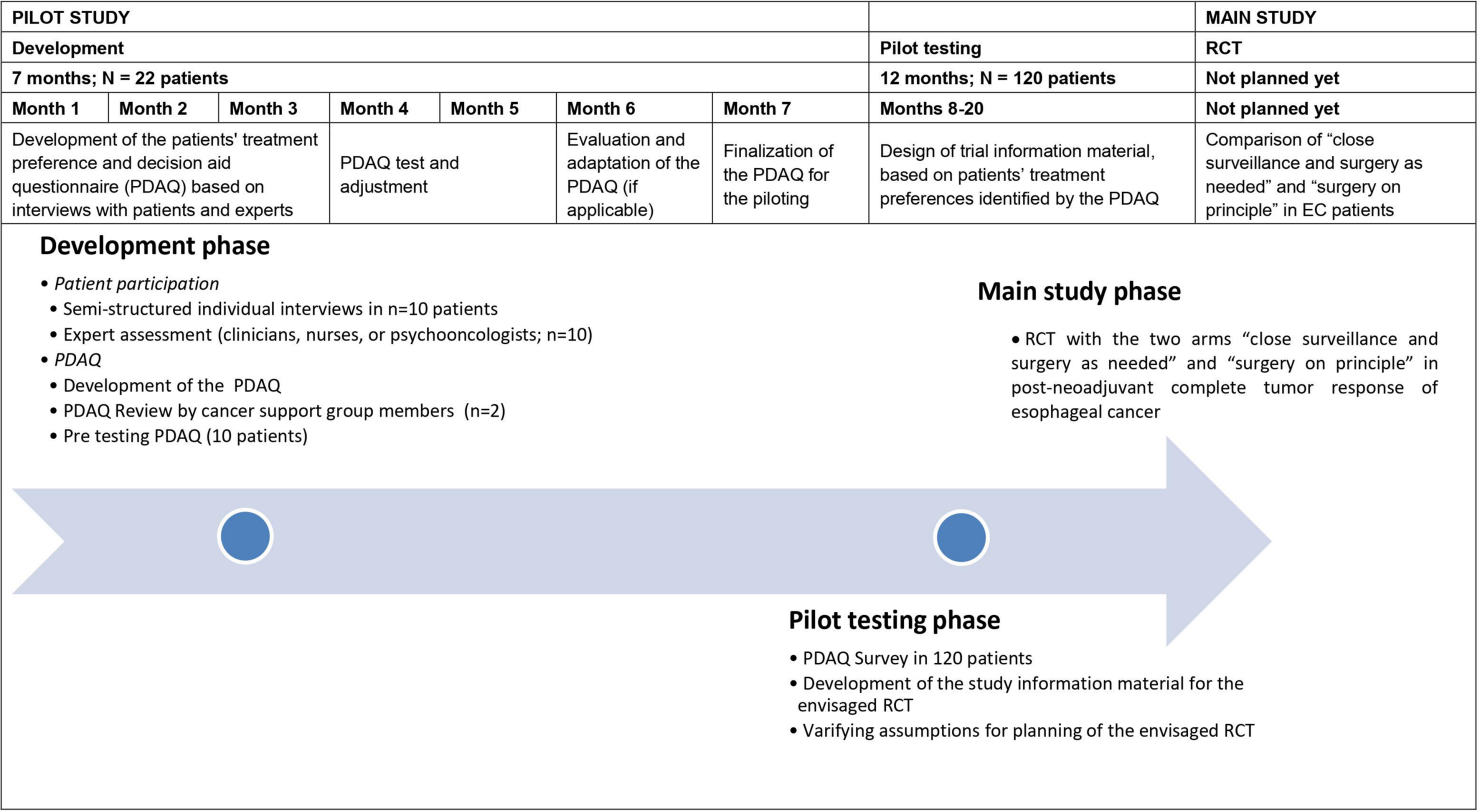
Flow Chart.

Based on the information regarding patients’ goals and attitudes, peer-support group members and medical experts’ attitudes, the final PDAQ will be constructed. In the subsequent pilot phase, *n* = 120 patients with EC after nCT or nCRT are asked to fill in the PDAQ in a multicenter trial in order to develop patient-centered trial information to serve as study material in the envisaged RCT. The specific study phases are depicted in **Table 1**. Furthermore, details about the study procedures of the pilot phase are provided in **Figure 1**.

**Table 1:** Study procedures.

Phase	Study Procedures
**Development phase (0–7 months)**	Interview guideline
	Patient eligibility
	Enrollment, study information, and informed consent
	Disease specific treatment data of study condition (EC)
	Interviews
	Patients in individual interviews (*n* = 10–15)
	Medical experts (*n* = 10)
	PDAQ
	Development
	Pre-testing (*n* = 10 patients; *n* = 2 patients advocates)
	Adaption
**Pilot phase (8–20 months)**	PDAQ Survey (*n* = 120 patients including)
	Age
	Gender
	Education
	FoP-Q-SF
	PHQ-9
	GAD-7
	EORTC-QoL-C30
	OES18
	Trial information material
	Disease specific treatment data of study condition (EC)

PDAQ, Preference and Decision Aid Questionnaire; FoP-Q-SF, Fear of Progression Questionnaire Short-Form; PHQ-9, Patient Health Questionnaire-9; GAD-7, Generalized Anxiety Disorder Screener-7; EORTC-QoL-C30, European Organization for Research and Treatment of Cancer’s Core Quality of Life Assessment; OES18, Oesophageal short module of the EORTC questionnaire.

### Participants

In the development phase patients’ screening for trial eligibility will be performed at the Medical Center University Freiburg. In the pilot phase the PDAQ survey will be performed multicentric in total five specialized centers in Germany. Eligible patients according to the inclusion criteria will be identified through medical records and will be patients after neoadjuvant treatment and before or after surgery. Patients, who participated in the pilot phase, will not participate in the consecutive RCT.

Patient participation in the study is voluntary and patients can withdraw their consent to participate at any time during the study without incurring disadvantages in treatment. Patients will be given sufficient time to read and understand the study information, to review the information, ask questions, and receive satisfactory answers from the trial physician. Subsequently, patients will be asked to sign the informed consent after completed information process. Patients eligible to participate in the study (development phase and pilot phase) have to fulfill the following inclusion criteria:

#### Inclusion Criteria

Diagnosis of non-metastatic EC, including both epidemiologically relevant histologies of EC esophageal adenocarcinoma (EAC) and adenosquamos carcinoma and squamous cell carcinoma (ESCC) according to the Universal Integrated Circuit Card (UICC) definition.

Scheduled or running treatment by western standard of care multimodal treatment schemes (nCT plus surgery and nCRT plus surgery).

- Age > 18 years.
- Patients before or after surgery.
- Ability to read and understand German.
- Willingness and ability to give informed consent before study entry.
- Patient’s written informed consent has been obtained.

#### Exclusion Criteria

Patients who meet the following exclusion criteria cannot participate in the trial:

- No written consent available.
- Patients with gastric cancer.
- Patients with tumors of the cervical esophagus.
- Co-morbidity with contraindication for esophageal surgery.
- Patient without legal capacity who is unable to understand the nature, significance, and consequences of the study.
- Concurrent medical or psychiatric condition that might preclude participation in the study according to investigator assessment.
- Cognitive or other type of impairment (such as severe psychiatric disorders and severe cognitive disorders that would interfere with completing paper-pencil questionnaires).
- Simultaneous participation in other studies which could interfere with this study and/or participation before the end of a required restriction period.

### Study Procedures

#### Development Phase

Patients’ will be asked to provide basic demographic data regarding age, gender, ethnicity, highest level of education, employment status, and marital status. Medical data such as current health status (Eastern Cooperative Oncology Group [ECOG] performance status), time since diagnosis, tumor type (adenocarcinoma, squamous cell carcinoma), clinical stage (cT/cN category), pathological stage (pT/pN category and tumor regression staging), and previous and actual medical treatment is taken from the medical documentation.

Based on patient preferences identified from literature review and expert opinion, relevant issues will be phrased for structuring the interviews.

For patients to be able to evaluate treatment options, they need to have adequate knowledge of treatment opportunities and realistic expectations of potential benefits and harms. Therefore, the interviews assessing patients’ treatment preferences, could start by repeating the information about both the “surveillance and surgery as needed” and the “surgery on principle” procedures including a detailed description of respective advantages and disadvantages. It should be stressed, that it is unknown which of the two procedures is superior and that the advantage of the “surveillance and surgery as needed” method is that post-treatment recovery might be quicker and less impairment of long-term HRQoL might be apparent. The advantage of the “surgery on principle” procedure might be improvement of local tumor control and, therefore, improved disease-free survival and the possibility for pathohistological examination of the surgical specimen (19).

Even though patients have no choice of treatment in an RCT, it is important to evaluate their expectations regarding treatment choice along with their constraints regarding RCT. Therefore, the interview guide includes patients’ concerns of clinical trials that might be:

- Feeling “left out” and cannot decide by myself which treatment I would like to choose (in a RCT)
- Feeling emotionally challenged in the expectation maybe not to be randomized in to my preferred treatment arm
- Feeling satisfied to be part of medical research that can help improving my or other patients’ treatment situation in the future
- I prefer taking part in a clinical trial, to be one of the first people to benefit from a new treatment, knowing there’s also a chance that the new treatment turns out to be no better, or worse, than the standard treatment

The interview will end with the question: “If you are randomly assigned to group A, would you accept the assignment or request a group change?”

The individual patient interviews will explore additionally patients’ need for information, their expectations regarding treatment, their subjective experiences, and their individual actions regarding decision making.

Surgeons/medical experts might also have treatment preferences that hinder the study information process and might contribute to recruitment bias. Therefore, medical experts/recruiters of the planned centers are asked regarding their attitudes towards both treatment options to ensure that they objectively inform patients about risks and benefits. Further the specialists are asked to focus on two basic questions: (a) are issues included in the preliminary study information material which they consider irrelevant for this patient group and if so, why do they consider these issues irrelevant? and (b) are there issues missing from this information material that the specialists consider relevant and if so, why do they consider these issues relevant?

A project team member with experience in qualitative research methods will conduct the interviews with patients and medical experts (i.e., 3–5 clinicians out of the field of EC treatment, 3–5 nurses, and psycho-oncologists) using the interview guides. Duration of the interviews are calculated approximately 30 minutes. Members from an adequate cancer support group will be asked to provide a review of those gathered views and opinions.

In the development phase, the qualitative analysis of the interviews will lead to a list of issues representing the insights of patients’ preferences towards treatment choice. Both, the qualitative analysis of the patients’ interviews (including former patients from the cancer support group) and the medical experts’ opinion will lead to an adaptation of issues, if applicable. A preliminary PDAQ questionnaire will be developed from the list of issues and 10 patients are asked to give their comments on the phrases using a think-aloud technique. These findings will lead to the final PDAQ with phrased items that can be evaluated for relevance and importance.

#### Pilot Phase

After pretesting the preliminary version of the PDAQ will be revised. The final PDAQ will assess patients’ needs, preferences, and its influencing factors towards the choices of treatment. The results of the questionnaire survey in the pilot testing phase will provide information for adapting the informed consent material to the patients’ needs and preferences for the main study. Patients’ information needs and values identified by the PDAQ will be transferred in the proven format of decision-support to be easy to understand, well-structured, clear, and helpful, which will serve as study information material in the envisaged RCT. The medical expert opinion as well as the review of former patients from the cancer support group are included in developing the study information material.

In the pilot phase, the survey includes the PDAQ questionnaire, captures sociodemographic and medical information and the following standardized instruments:

- FoP-Q-SF: The Fear of Progression Questionnaire Short-Form [FoP-Q-SF (20)] is a concise standardized psychological instrument to measure the fear of progression (FoP) in chronically ill patients (cancer, rheumatic diseases, and diabetes mellitus). The questionnaire consists of 12 items and covers the factors: *affective reactions*, *partnership/family*, *occupation*, and *loss of autonomy*.
- PHQ-9: The Patient Health Questionnaire-9 [PHQ-9 (21)] is a brief and validated measure of depression severity. It consists of 9 items and covers the 9 depression criteria of the Diagnostic and Statistical Manual of Mental Disorder IV (DSM-IV). For each item, the patient has to choose from 0 (“not at all”) to 3 (“nearly every day”). Thus, the maximum score is 27.
- GAD-7: The Generalized Anxiety Disorder Screener-7 [GAD-7 (22)] is a standardized 7-item self-report anxiety questionnaire assessing the anxiety symptoms: *nervousness*, *inability to stop worrying*, *excessive worry*, *restlessness*, *difficulty in relaxing*, *easy irritation*, and *fear of something awful happening*. Similar to the PHQ-9, the total score is calculated by adding together the scores for all items ranging from 0 (“not at all”) to 3 (“nearly every day”).
- EORTC-QoL-C30: The European Organization for Research and Treatment of Cancer’s (EORTC) core quality of life assessment [EORTC-QoL-C30 (23, 24)] is a validated instrument to assess the quality of life of oncological patients. It contains 30 questions in 10 subscales. Furthermore, the EORTC-QoL-C30 has a specific module for EC called OES18 (25) to assess the detailed symptoms of EC-patients.

The survey will be conducted in collaboration with five centers. A final sample of n = 120 patients will be included.

### Trial Information Material

Based on the results of the survey, the information material for the envisaged RCT will be revised. In addition, a check-list for clinicians will be developed to guide the information process.

Recommendations for the design of risk information include graphical displays to increase the effectiveness of risk communication (3); therein simple bar charts are preferred and absolute risks are given rather than relative risks, and comparisons with everyday risks are proposed.

We will base the development of information material on useful design formats that follow the quality standards for patient decision aids for presenting risk information and prediction models [i.e., the SUNDAE (Standards for Universal Reporting of Patient Decision Aid Evaluation) checklist (26), and the IPDAS (International Patient Decision Aid Standard) Collaboration] that include percentiles, ratios, and pictographs (7, 27, 28).

Estimates for OS after nCRT or nCT + esophagectomy can be obtained from an interactive web-based instrument (nomogram), where the individual survival of patients is estimated based on their individual pathological, demographic, and treatment data (29).

It is suggested that clinicians have a toolbox of presentation styles to suit different patients and outcomes (30). It may be that multimodal consultations, incorporating verbal and visual information, presented differently, such as event rates, risk ladders, or bar charts, may maximize patient understanding of different treatment outcomes. Option grid formats can be used to display attributes of treatment options and to answer patients’ most relevant questions (16, 17, 31). Patient-relevant questions when making trade-offs with regard to their treatment decision might be for example:

How long will I stay in hospital? (Risk of in hospital mortality)
Which treatment is the best for long-term survival? (5-year survival rate)
What are the chances of cancer coming back in the esophagus? (Risk of relapse)
How long will it take to recover? (Risk of persistent gastrointestinal problems)
How many patients experience physical side effects (e.g., speech pathologies, dysphagia, respiratory restrictions, pain, anxiety, etc.)? (Risk of post-treatment complications)
How many consultations will I have? (Burden of appointments)

The trial information material developed and designed during development and pilot testing phase including a clinician’s check-list to guide the information process will be used to provide comprehensive education and information about the randomized controlled trial for patients with non-metastatic EC after nCRT.

### Data Management and Monitoring

During the study, all personal data will be kept separately in the patient identification log (identification of patient and contact details). All patient data will be captured in pseudonymized form. After transcription all audio files will be stored until the end of the project at least for three years and then be deleted. The data management will be performed with REDCap™ Version 9 (redcap@vanderbilt.edu).

Details on data management (procedures, responsibilities, deviations, etc.) will be described in a data management manual which will be continuously updated and maintained during the trial. The technical specifications of the database will be described in a data description plan (DDP). Before any data entry is performed, the trial database and electronic case report forms (eCRFs) will be validated. Site data entry personnel will not be given access to the trial data base until they have been trained and signed an access form.

Statistical Analysis System (SAS) software will be used to review the data for completeness, consistency and plausibility. The checks to be programmed will be specified beforehand in a data validation plan. After running the check programs, the resulting queries will be sent to the investigator for correction or verification of the documented data. Data corrections will be entered directly into REDCap by the responsible investigator, or designated person. Query forms which contain the corrections must be confirmed by the dated signature of the investigator (not the study nurse) in the designated places. Due to the characteristics of the study, no data monitoring committee (DMC) will be included.

### Biostatistical Planning and Analysis

#### Development Phase

In this phase the focus lies on qualitative analyses of the patient interviews supported by specific software (MAXQDA). Statistical analyses are confined to descriptive analyses. All qualitative analyses will be performed at the Endowed Professorship Self-help Research, Interdisciplinary Tumor Center Medical University Freiburg, with the support of the Clinical trial unit if applicable.

#### Pilot Phase

The pilot phase mainly has two objectives:

1) The development of patient-centered information material optimally fulfilling the individual needs of the patients with the aim to improve the recruitment of patients for the clinical trial and to improve their consent to randomization between the standard treatment “surgery on principle” and the experimental treatment “surveillance and surgery as needed” and their adherence to the randomized treatment.

2) To verify assumptions for sample size calculation for the planned RCT regarding the rate of EAC vs. ESCC, the rate of nCRT vs. nCT, and the pCR rate after surgery.

With regard to objective 1) the effects of socio-demographic, disease-specific, and psycho-social factors on patients’ treatment preference (“surgery on principle” versus “surveillance and surgery as needed”) will be analyzed. Additionally to descriptive analyses, univariate and multivariate logistic regression models will be used to identify which factors may be associated with patients’ treatment preference. Effect sizes will be quantified by odds ratios with 95%-confidence intervals and tested using the Wald test. P-values will be interpreted in a descriptive sense. Those factors identified as influential with a relevant effect size will then especially be considered in the development of the patient-centered trial information material and check-list for clinicians in the planned RCT.

Sample size was chosen based on feasibility without formal sample size planning based on statistical power calculations. The inclusion of 120 patients from 5 clinical centers within a time period of 6 to 12 months was regarded as feasible. The following statistical consideration only exemplifies the possible conclusions with the chosen sample size: 120 patients would provide 80% power for an identification of a factor influencing patients’ preference with an odds ratio of 3 at a significance level of.05, considering adjustment for other correlated factors (variance inflation factor 1.2). With regard to objective 2) descriptive analyses of tumor type, type of neoadjuvant therapy, and of pCR status after surgery will be performed.

## Discussion

Due to evidence of a possible equivalence with regard to OS of the two treatment options “surveillance and surgery as needed” and “surgery on principle” in patients with post-neoadjuvant complete response of EC, this pilot study is aimed to involve patients early in the development of the main trial. Hereby integration of patient’s needs and preferences, and optimization of patients’ information and inclusion in the planned RCT should be achieved. We use a mixed-methods approach in the concept development and the pilot testing phase of the study.

In our experience patients are interested to be involved in clinical trials and other psychosocial studies. Against the background of an increasing demand for patient participation in clinical trials this study realizes an innovative patient-centered approach to involve patients and patients’ mandatories in various stages of the clinical trial. Patients may benefit in participating in the study by helping to create comprehensive study information material and to optimize patient care. Patient’s mandatories may help to improve the study material from a patient’s perspective. The risks for patients and medical experts in this interview and questionnaire survey are estimated to be very low.

Nevertheless, participation in a patients’ evaluation (i.e., interviews and questionnaire survey) to assess treatment preferences regarding both a standard principle surgery therapy and an experimental treatment with active surveillance and surgery as needed might bear the risk that patients get new information towards treatment options they did not have before in their own treatment. Patients might get emotionally affected when they recognize they would have preferred another treatment as they received.

Considering the clinical relevance of identifying factors influencing patients’ decision towards one treatment option, it is important to mention specific problems with adherence to the allocated trial treatment in completed RCTs comparing surveillance with surgery on principle in EC patients (17, 32, 33). In the published trials, a striking difference in the compliance to the allocated treatment was to be noticed between the different arms of the trials, with higher rates of non-compliance to the protocol in the surgical-arms. For the ongoing SANO-trial, this factor was included to the study design by using a cluster-randomisation (13). For the planned RCT, we are going to address this issue not alone by specific trial design but also by conducting this pilot study to create patient-centered trial information material. The eligibility criteria of the main trial won`t be affected by the results of the patient’s participation study. The study aims to optimize information material for the main trail we expect that this shall improve study recruitment and protocol adherence by creating comprehensive and patient-centred study information material.

### Ethics and Dissemination

The research will be conducted in accordance with the principles of Good Clinical Practice. The study is registered at the German Clinical Trials Register (DRKS00022050, October 15, 2020) and has been approved by the Medical Ethical Committee of Freiburg University Medical Center (No. 20-1037). Any amendments to the protocol will be communicated and re-approved by the ethics committee. The findings of this study will be disseminated widely through peer-reviewed publications and international conference presentations.

## Data Availability Statement

The original contributions presented in the study are included in the article/supplementary material. Further inquiries can be directed to the corresponding author.

## Ethics Statement

The studies involving human participants were reviewed and approved by Medical Ethical Committee of Freiburg, University Medical Center. The patients/participants provided their written informed consent to participate in this study.

## Author Contributions

Primary sponsor: JHo. Conceptualization: JW, AK, and JHo. Project planning: JW, AK, CS, JHo, JHi, and MC. Writing: AK, MC, and JW. Statistical counseling: CS. Funding acquisition: JW, JHo, and JHi. Editing: JW, AK, CS, JHo, MC, MR, PG, CB, and JHi. All authors provided review of the manuscript. All authors read and approved the final manuscript.

## Funding

This study is supported by a research grant (grant number 01KD1908) “Nationale Dekade gegen Krebs” provided by the Federal Ministry of Education and Research (BMBF). The study design has been peer-reviewed and approved by the funding body. The funder was not involved in the development of the protocol. The funder did not influence the study design and will not take part in data collection, analysis and interpretation or in writing the manuscript. The Funder monitors the study to ensure that legal requirements regarding the use of funds and patient safety or the security of data are complied with and that they are made available to the public in a transparent manner. Otherwise the funder has no influence on the study. The article processing charge was funded by the Baden-Wuerttemberg Ministry of Science, Research and Art and the University of Freiburg in the funding program Open Access Publishing.

## Conflict of Interest

The authors declare that the research was conducted in the absence of any commercial or financial relationships that could be construed as a potential conflict of interest.

## Publisher’s Note

All claims expressed in this article are solely those of the authors and do not necessarily represent those of their affiliated organizations, or those of the publisher, the editors and the reviewers. Any product that may be evaluated in this article, or claim that may be made by its manufacturer, is not guaranteed or endorsed by the publisher.
